# Geospatial disparities in post-pandemic SARS-CoV-2 vaccination: Evidence from Chile beyond the national success

**DOI:** 10.1371/journal.pgph.0006808

**Published:** 2026-07-27

**Authors:** Muriel Ramírez-Santana, Juan Correa, Paola Rubilar, Mauricio Apablaza, Luis Canales, Loreto Nuñez-Franz

**Affiliations:** 1 Departamento de Salud Pública, Facultad de Medicina, Universidad Católica del Norte, Coquimbo, Chile; 2 Escuela de Arquitectura, Universidad Gabriela Mistral, Santiago, Chile; 3 Doctorado en Geografía, Pontificia Universidad Católica de Chile, Santiago, Chile; 4 Centro de Epidemiología y Políticas de Salud, Facultad de Medicina Clínica Alemana Universidad del Desarrollo, Santiago, Chile; 5 Facultad de Gobierno, Universidad del Desarrollo, Santiago, Chile; 6 Facultad de Economía y Negocios, Universidad de Talca, Talca, Chile; 7 Departamento de Salud Pública, Facultad de Ciencias de la Salud, Universidad de Talca, Talca, Chile; University of Michigan, UNITED STATES OF AMERICA

## Abstract

While Chile achieved one of the world’s highest and most equitable SARS-CoV-2 vaccination coverages during the pandemic, less is known about the post-pandemic stage. This study examines within-country geospatial disparities in COVID-19 vaccination uptake and their socio-demographic determinants in two urban areas of Chile. A population-based survey conducted in May 2024 collected individual data on vaccination status and socio-demographic characteristics. These were georeferenced and linked to census information at the household block level. Spatial analyses explored clustering and correlations between the number of vaccine doses and variables such as age structure, educational attainment, overcrowding, and proximity to public health facilities. Moran’s Index was used to assess spatial autocorrelation and bivariate relationships, disaggregated by sex and city. The number of vaccine doses inoculated displayed significant spatial concentration, particularly among women. A positive spatial correlation was found between vaccination and higher education, and a negative correlation with overcrowding in one city. Distance to health facilities was not significantly associated with vaccine uptake. Across both cities, a higher proportion of older adults was associated with higher vaccination rates, with gender-specific patterns observed. Despite Chile’s previously homogeneous vaccination performance, post-pandemic findings reveal emerging socio-spatial disparities. The return of demographic and territorial determinants suggests a shift toward structural influences on vaccine uptake. These results underscore the need for territorially tailored public health strategies to reduce intra-country disparities in immunization access over time.

## Introduction

The influence of geographical conditions on the distribution of diseases and health interventions, such as vaccinations, is well known [1–9]. Spatial disparities can shape both disease distribution and access to preventive measures, of which vaccination is one of the most important strategies for controlling infectious diseases [10]Several studies have focused on describing the spatial distribution and geographical determinants related to vaccination coverage and vaccine access worldwide [1–9]. Some factors identified as influencing general vaccination distribution in territories are socioeconomic status [8,11,12], car ownership [3,13], access to health facilities [5,9], health status [14], population density [3,15], ethnic diversity [12,16], educational level [1,5,16], rurality [17], religion [5], employment [18], sex [19], age, and insurance status [12,20].

However, studies on the spatial distribution and territorial determinants of vaccination coverage in the post-pandemic phase of SARS-CoV-2 are scarce [6,12,14,21], especially in Latin America. Understanding how vaccination patterns evolve beyond the emergency phase is crucial for designing new policies to ensure equitable immunization coverage, particularly as public health priorities shift and risk perceptions decline. Specifically, during COVID-19, the epidemiological situation was a key driver of vaccination [21]. Thus, once the pandemic was over, in August 2023 [22], risk perception decreased, and motivation to be vaccinated declined, resulting in lower vaccination coverage.

Chile represents a compelling case study for examining vaccination dynamics in the post-SARS-CoV-2 era. During the first year of the COVID-19 pandemic in Chile, a social disparity was observed in the territorial distribution of immunity (before vaccination), identifying clusters of infection in the most vulnerable neighborhoods [23,24]. Subsequently, with the implementation of vaccination, these geographical disparities of immunity were reduced. Vaccination was uniformly distributed across the territories thanks to a broad, unrestricted access campaign [25].

Following the WHO recommendations [26], since 2023, the Chilean Ministry of Health has decided to include the vaccine against COVID-19 in the national vaccination schedule, focusing on specific population groups who are at higher risk of developing severe disease [27]. Vaccines against SARS-CoV-2 were still necessary for individuals at risk of serious disease in 2024. The elderly and those with chronic conditions remain particularly vulnerable to severe outcomes. Additionally, the emergence of new variants and vaccine escape mechanisms justifies the continuation of vaccination campaigns, in line with the recommendations of Chilean health authorities. However, it has been challenging to maintain sufficient vaccination coverage lately. As the pandemic progressed and the urgency regarding vaccination declined, vaccination coverage has also been deteriorating and may be affected by socio-demographic determinants [28].

In a multidimensional study of the spatial process of COVID-19 vaccination in the United States, Yang argues that the spatial processes underlying vaccination coverage are essentially local [13]. Thus, outreach strategies need to be adjusted to achieve the objective. Helpful information is imperative for tailoring vaccination strategies and public messages [29]

This study aims to identify the geospatial distribution of post-pandemic SARS-CoV-2 vaccination in two Chilean cities and explore the socio-demographic factors influencing vaccine uptake, disaggregated by sex. The underlying hypothesis is that, once the epidemic ended, socio-demographic factors shaped access to vaccination, resulting in a heterogeneous geographical distribution of immunization.

## Materials and methods

### Study design

This is a population-based cross-sectional study. From 02/05/2024 to 31/05/2024, the recruitment and fieldwork phase of a four-round population-based COVID-19 seroprevalence study was conducted in Talca and the La Serena/Coquimbo conurbation in Chile. The present study and three previous [23,28] were conducted by researchers affiliated with three universities, two of which are located in the cities selected for fieldwork. City selection follows a convenience sampling approach, reflecting the researchers´ institutional locations and their established relationships with local health and community networks.

### Participants and setting

**Fig 1:**
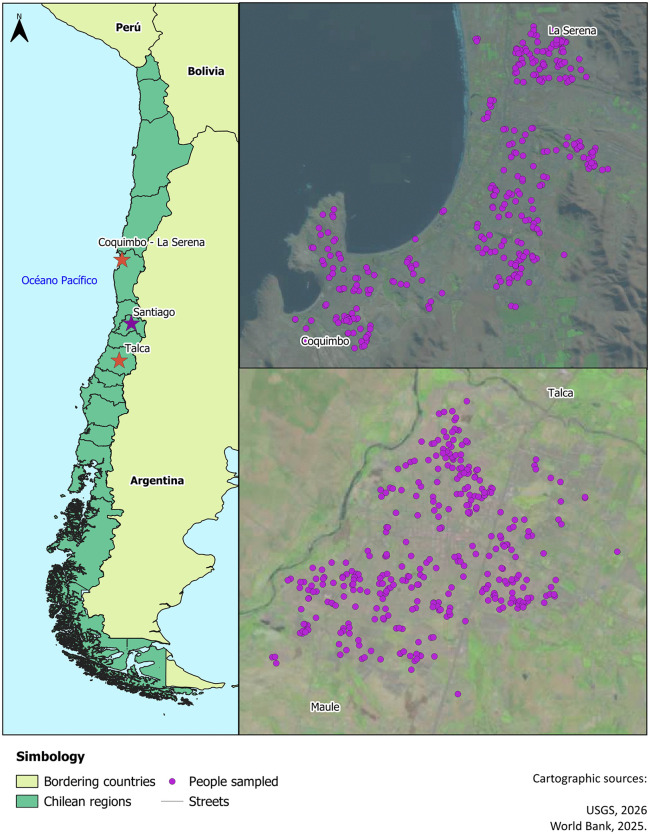
Map of Chile and survey locations. Source: Designed by the authors. Regional and communal administrative boundaries were sourced from the World Bank Official Boundaries dataset (World Bank, 2025; https://datacatalog.worldbank.org/search/dataset/0038272/world-bank-official-boundaries), licensed under CC BY 4.0 (https://datacatalog.worldbank.org/public-licenses?fragment=cc). Satellite images were sourced from the United States Geological Survey EarthExplorer platform (USGS, 2026: https://earthexplorer.usgs.gov/), which are **U.**S. Public Domain (https://www.usgs.gov/information-policies-and-instructions/copyrights-and-credits). Attribution is displayed in the lower right corner of each map. No proprietary mapping services were used.

The La Serena/Coquimbo conurbation is located 450 kilometers from Santiago (the country’s capital) and Talca, 250 kilometers south of the capital. Serum samples were tested for antibodies against SARS-CoV-2 at a university laboratory in Santiago (Fig 1). Stratified two-stage random sampling by census district was carried out. First, a block random selection, and second, a household selection using systematic skip. Within the household, all members aged 7 years or older were invited to participate, without exclusion. Details of the methodology and participant selection can be found in Nuñez-Franz et al. (2025) [28].

A total of 654 people were enrolled. The sample of participants proved to be representative and evenly distributed in both cities. Spaces without participants in the cities correspond to uninhabited sectors, designated for agricultural or commercial use. These areas were identified using digital mapping from the Chilean Instituto Nacional de Estadísticas*,* generated for the population and housing census, which digitized only blocks with more than five residents and excluded non-residential areas [30]. This was observed especially in the city center of Talca.

### Variables

The **dependent variable** corresponds to each case’s total number of vaccine doses, while the **independent variables** are territorial factors. For this, each case was geo-referenced to its census block [31] and subsequently assigned the socio-demographic information of its census tract, such as age, education, overcrowding, and access to health facilities. The proportion of older adults (aged sixty-five and over) was considered for age. The educational level was analyzed as the percentage of the population with a higher level of education (higher technical or university, postgraduate). For overcrowding, the rate of households where 2.5 or more people share a bedroom was used [32,33], and access to healthcare was operationalized as the distance to the nearest health center (Table 1).

**Table 1:** Study Variables: Description, Classification, and Sources.

Variable	Description	Type of variable	Source of data
Sex	Sex of the selected person (Male/Female).	Categoric nominal	Collected by trained health personnel in a questionnaire.
Age	Age of the selected person (18 – 99).	Continue	Collected by trained health personnel in a questionnaire
Vaccine doses	Total number of vaccine doses received by the selected participants.	Discrete	Obtain from the National Immunization Registry, or collected by trained health personnel.
Age (elderly people)	Corresponds to the proportion of older adults (aged sixty-five and over) for each census block.	Continuous	Census 2017
Education	Corresponds to the percentage of the population with a higher level of education (higher technical or university, postgraduate) for each census block.	Continuous	Census 2017
Overcrowding	Corresponds to the rate of households where 2.5 or more people share a bedroom. This standard is defined by the National Statistics Institute (Instituto Nacional de Estadísticas, 2024) and Ministerio de Desarrollo Social (2024)	Continuous	Census 2017
Access to health facilities	Corresponds to the distance to the nearest health center. This was determined by calculating the Euclidean distance from each census block to the nearest health center using the software QGIS	Continuous	Census 2017 and Chilean Ministry of Health 2024

### Data sources and measurements

Individual variables (sex and age) and the number of SARS-CoV-2 vaccine doses were collected via a questionnaire completed by trained health personnel. Vaccination status was verified using a two-tier approach: (1) participants provided documentation by accessing their records through the national immunization registry portal or presenting vaccination cards; (2) when this was not feasible, participants authorized the research team to obtain records directly from the national immunization registry. Vaccination status was classified by the number of vaccine doses received as of the study date.

### Statistical analysis

Spatial analysis was performed on the total number of samples from each city, separated by sex, to account for potential differences in vaccination uptake between males and females, and calls in the literature for sex-disaggregated reporting to inform more equitable vaccination programs [19,34]. A spatial autocorrelation analysis of the variable total doses was first developed for each sample (total city sample and by sex) using Moran’s Index. Moran’s spatial autocorrelation index was used because it better detects areas of concentration or dispersion across a region, whereas other indicators (such as Geary or Tango) focus more on identifying spatial outliers [35]. The index ranges from -1–1 for the statistical significance test. When the index value is positive, it indicates greater spatial concentration. Conversely, a negative value indicates greater spatial dispersion. At the same time, values closer to zero indicate homogeneity, and values closer to 1 indicate clustering. Secondly, a bivariate spatial autocorrelation analysis (Local Moran’s Index Bivariate) was conducted to assess the spatial correlation between variables [36]. For further details and formulas, see Supplementary Material Statistical analysis (S1 Appendix. Statistical analysis details and formulas). Online open software QGIS and GEODA were used to perform the analysis.

### Ethical consideration

The study was conducted following the Declaration of Helsinki, and the study protocol was reviewed and approved by three independent Scientific Ethical Committees: 1) Scientific ethical committee from the Faculty of Medicine, Universidad Católica del Norte, Resolution number 63/2023, dated October 16th, 2023. 2) Scientific ethical committee from the Faculty of Medicine, Universidad del Desarrollo, dated December 13th, 2023, and 3) Scientific ethical committee from Universidad de Talca, Folio 30–2023-E, dated April 17th, 2024. Additionally, it was approved by the Institutional Committee of Biosecurity of Universidad Católica del Norte 07/2023, dated October 2023, Universidad del Desarrollo, CIB-FORM-01B, dated 14 November 2023, and Universidad de Talca 20-CBS-2023 dated November 9th, 2023. Written informed consent was obtained from all subjects involved in the study. For participants under 18 years old, an assent form was signed by the minor after their guardian signed a consent form.

## Results

### Participants description

Out of a total of 654 participants, 318 were from the La Serena/Coquimbo conurbation, and 336 were from Talca. The sample consists mainly of women aged over 40 with completed schooling. At the time of the study, most people had had an episode of COVID-19 and had received at least 2 vaccine doses. Vaccine data were obtained mainly from the National Immunization Registry (96%), the remaining records were obtained through the Patient Portal of the Chilean National Immunization Registry. The description of the main variables in the populations studied is presented in Table 2.

**Table 2:** Description of the main variables studied in terms of the city: Seroprevalence study 2024.

Variable	Category	La Serena/Coquimbo (n = 318)		Talca (n = 336)	
		n	%	n	%
Sex	Women	204	64.1	218	64.9
	Men	114	35.9	118	35.1
Age	19 and less	18	5.7	7	2.1
	20-39	72	22.6	68	20.2
	40-59	112	35.2	114	33.9
	60 and more	116	36.5	147	43.8
Education	Basic or less	38	12.5	59	17.8
18 years and over	Highschool	161	53.0	158	47.7
	Technician	38	12.5	43	13.0
(n = 635)	Professional or postgraduate	67	22.0	71	21.5
COVID-19 episodes (n = 277)	Once	94	77.7	130	83.3
	Twice	21	17.3	20	12.8
	Three times	6	5.0	6	3.9
Vaccination history	No vaccination	9	2.8	2	0.6
	Basic scheme* and/or boosters (2020–2022)	182	57.2	145	43.1
	Bivalent** and/or Omicron (2023–2024)	127	39.9	189	56.3

*2 doses of vaccines administered at least 28 days apart (Sinovac, Pfizer-BioNTech, AstraZeneca/Oxford).

**Pfizer-BioNTech COVID-19 Original/Omicron BA.4/BA.5 (bivalent) vaccine.

### Cluster analysis in Talca

**Fig 2:**
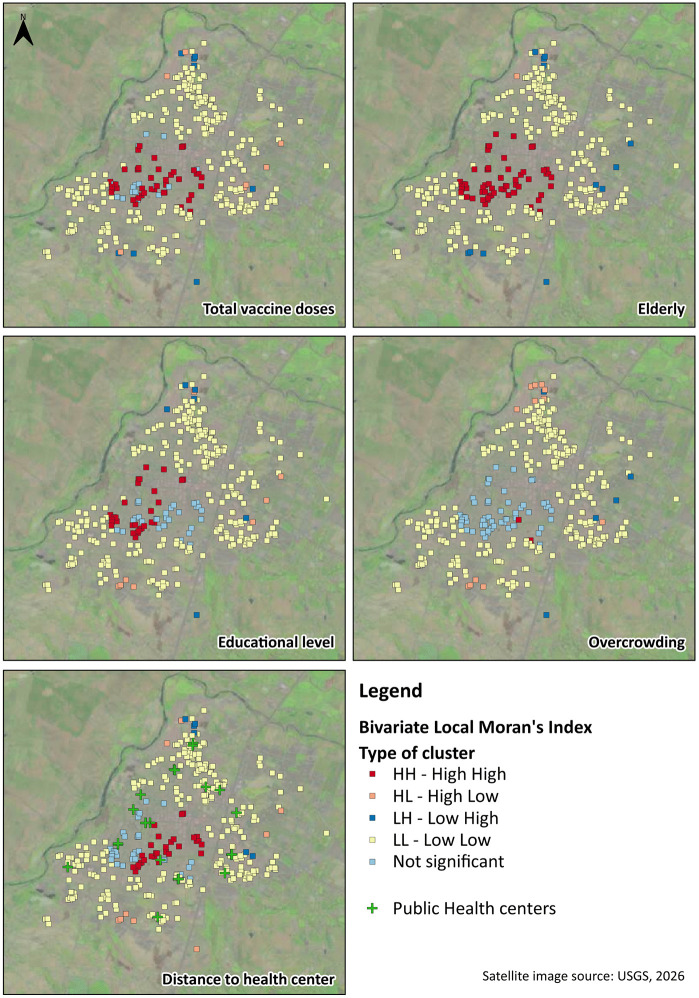
Map of the city of Talca showing the distribution of vaccine dose clustering and its associations with socio-demographic factors (age over 65, educational level, overcrowding, and distance to health center). Source: Designed by the authors with data from the study. Regional boundaries were obtained from the World Bank Official Boundaries dataset (https://datacatalog.worldbank.org/search/dataset/0038272/world-bank-official-boundaries). CC-BY 4.0 license (https://datacatalog.worldbank.org/public-licenses?fragment=cc). Satellite images were sourced from the United States Geological Survey EarthExplorer platform (USGS, 2026: https://earthexplorer.usgs.gov/), which are **U.**S. Public Domain (https://www.usgs.gov/information-policies-and-instructions/copyrights-and-credits). Attribution is displayed in the lower right corner of each map. No proprietary mapping services were used.

In Talca, a cluster of higher vaccine numbers is observed in the central sector of the city (Fig 2), where the highest proportion of older adults, higher educational levels, and lower overcrowding are also concentrated. However, these last two variables are not significant in the statistical analysis (Table 3). The same observations are replicated when analyzing women separately (Table 3 and S1 Fig). However, no significance is observed in the geographic distribution patterns for any of the analyzed variables in the male population (Table 3 and S2 Fig).

**Table 3:** Spatial autocorrelation analysis for all population and by sex, Talca.

Variable	Moran’s Index	Expected index	Z score	P Value	Conclusion
**Total sample**					
Number of doses	0.208	0.003	2.831	0.010	Slight spatial association, significant
Elderly	0.092	-0.003	13.360	0.010	Slight spatial association, significant
Educational level	0.046	-0.003	6.697	0.001	No spatial association
Overcrowding	0.072	-0.003	-11.115	0.001	No spatial association
Distance to health center	0.005	-0.003	0.591	0.265	No spatial association
**Women**					
Number of doses	0.158	0.005	2.763	0.014	Slight spatial association, significant
Elderly	0.113	-0.005	10.055	0.001	Slight spatial association, significant
Educational level	0.031	-0.005	3.108	0.001	No spatial association
Overcrowding	-0.091	-0.005	-8.360	0.001	No spatial association
Distance to health center	-0.043	-0.005	-4.094	0.001	No spatial association
**Men**					
Number of doses	-0.019	-0.008	-0.443	0.349	No spatial association
Elderly	0.054	-0.008	2.887	0.007	No spatial association
Educational level	0.012	-0.008	0.660	0.245	No spatial association
Overcrowding	-0.031	-0.008	-1.623	0.461	No spatial association
Distance to health center	-0.005	-0.008	0.186	0.427	No spatial association

### Cluster analysis in La Serena/Coquimbo

**Fig 3:**
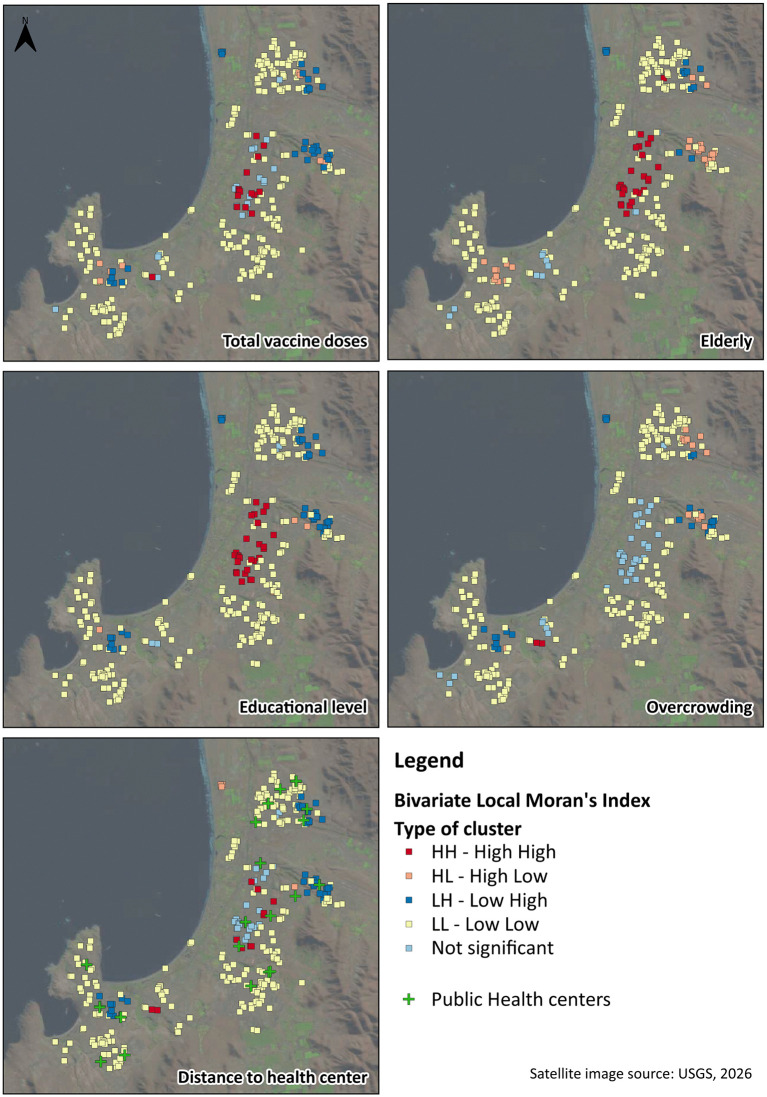
Map of the La Serena/Coquimbo conurbation showing the distribution of vaccine dose clustering and its associations with socio-demographic factors (age over 65, educational level, overcrowding, and distance to health center). Source: Designed by the authors with data from the study. Regional boundaries were obtained from the World Bank Official Boundaries dataset (https://datacatalog.worldbank.org/search/dataset/0038272/world-bank-official-boundaries). CC-BY 4.0 license (https://datacatalog.worldbank.org/public-licenses?fragment=cc). Satellite images were sourced from the United States Geological Survey EarthExplorer platform (USGS, 2026: https://earthexplorer.usgs.gov/), which are **U.**S. Public Domain (https://www.usgs.gov/information-policies-and-instructions/copyrights-and-credits). Attribution is displayed in the lower right corner of each map. No proprietary mapping services were used.Table 5 summarizes the presence and absence of spatial autocorrelation for vaccine doses and bivariate correlation between vaccination and territorial variables, according to the sample type (total sample, women, and men). The difference observed between the cities is notable. The number of doses shows spatial concentration across both towns and the female samples. The distribution of older people correlates significantly with the number of doses in the Talca sample, among women in that city, and among men in the La Serena-Coquimbo conurbation. Only in that context is there a significant spatial correlation between education level and vaccination in the total sample and across both sexes. At the same time, overcrowding is spatially correlated with immunization only in the male sample of the conurbation.

In La Serena/Coquimbo, a cluster of higher vaccination doses is observed in the central sector of the city, which coincides with a higher proportion of more educated people, a higher proportion of older adults, and less overcrowding (Fig 3). Nevertheless, these last two variables are not significant in the statistical analysis (Table 4). The same observations are repeated when analyzing women separately (Table 4 and S3 Fig). Concerning men, the factors associated with higher vaccination rates are higher levels of schooling, a higher proportion of older adults, and overcrowding (Table 4 and S4 Fig). These last two variables were non-significant in the La Serena/Coquimbo sample and among women.

**Table 4:** Spatial autocorrelation analysis for all population and by sex, La Serena/Coquimbo conurbation.

Variable	Moran’s Index	Expected index	Z score	P Value	Conclusion
**Total sample**					
Number of doses	0.114	-0.003	4.458	0.001	Slight spatial association, significant
Elderly	0.055	-0.003	3.375	0.001	No spatial association
Educational level	0.143	-0.003	9.001	0.001	Slight spatial association, significant
Overcrowding	0.084	-0.003	-5.391	0.001	No spatial association
Distance to health center	0.066	-0.003	4.247	0.001	No spatial association
**Women**					
Number of doses	0.120	-0.005	3.739	0.002	Slight spatial association, significant
Elderly	0.006	-0.005	0.265	0.385	No spatial association
Educational level	0.134	-0.005	5.197	0.001	Slight spatial association, significant
Overcrowding	0.066	0.005	-2.598	0.009	No spatial association
Distance to health center	0.054	-0.005	2.168	0.017	No spatial association
**Men**					
Number of doses	0.052	-0.009	1.148	0.126	No spatial association
Elderly	0.129	-0.009	3.235	0.002	Slight spatial association, significant
Educational level	0.135	-0.009	-3.593	0.002	Slight spatial association, significant
Overcrowding	-0.144	-0.009	-2.598	0.009	Slight spatial association, significant
Distance to health center	0.064	-0.009	1.168	0.017	No spatial association

**Table 5:** Presence of spatial correlation by variable and type of sample.

Variable	Talca total sample	Talca women	Talca men	La Serena/Coquimbo total sample	La Serena/ Coquimbo women	La Serena/ Coquimbo men
Number of doses	Yes	Yes	No	Yes	Yes	No
Elderly	Yes	Yes	No	No	No	Yes
Educational level	No	No	No	Yes	Yes	Yes
Overcrowding	No	No	No	No	No	Yes
Distance to a health center	No	No	No	No	No	No

## Discussion

The study aimed to identify geospatial vaccination distribution and factors related to post-pandemic SARS-CoV-2 vaccination in two Chilean cities. The findings show that there is some heterogeneity in the geospatial distribution of vaccination, while also confirming the hypothesis that this observation is related to socio-demographic factors in both cities. This contrasts with the homogeneous distribution previously observed in 2022 [25].

This study highlights how socio-demographic factors shape access to vaccination in the post-pandemic period, even in a country that initially achieved widespread coverage. In both cities analyzed, the high-high (HH) clusters of vaccine doses correspond to areas of lower socio-material vulnerability and the low-low (LL) clusters to areas of higher vulnerability, as evidenced by the Territorial Socio-Material Index of the UC Cities Observatory [37]. Generally, in areas of lower vulnerability, there is a higher average schooling, better housing materiality, and lower rates of overcrowding, while in areas of high vulnerability, there is less access to education and higher rates of overcrowding.

Systematic reviews and meta-analyses have reported COVID-19 vaccine hesitancy rates ranging from 42% (in Africa) to 16% (in Europe) [38], with acceptance rates also varying by country and continent, ranging from 13% (Iraq) to 92% (Tunisia) [39]. Various factors influence vaccine acceptance or hesitancy. For example, a county-level study conducted in the United States attributed 79% of the variation in vaccination rates to spatial patterns. The study shows that per capita income and the percentage of minorities have positive effects on vaccination rates, while age, mobile homes, and the uninsured population have negative effects on vaccine uptake [20]. A South African study using geo-additive models revealed geographically varying levels of vaccine hesitancy, with strong geospatial correlations between such hesitancy and beliefs [40]. Several factors associated with COVID-19 vaccine hesitancy have been identified in both conventional and geospatial studies [40,41]. One of the most significant is trust—both in the vaccine itself and in governments—where health and vaccine literacy play a key role [42–46]. Common to most of the studies reviewed is the recommendation to adapt vaccination strategies to local contexts.

In this study, Chile recorded COVID-19 vaccine acceptance rates as high as 98% at the start of the pandemic vaccination program, which subsequently declined over three years. Motivation to vaccinate and targeting COVID-19 vaccination to at-risk groups also influence the observed change over time. In this way, the study corroborates the suggestions of other researchers regarding the need to design vaccination strategies based on territorial vaccination surveillance [47], as these strategies have a greater impact on achieving high coverage [48].

Furthermore, the study contributes to the broader discussion on how vaccination campaigns should shift from emergency responses to long-term public health strategies across different global contexts, particularly in Latin America. Once the epidemiological emergency is over, socio-demographic factors such as age, sex, and education resume their influence on access to vaccination. In both cities, immunization exhibits central clustering, which may be partially explained by the higher concentration of the elderly in the center of the cities. Age was indeed a variable related to spatial distribution, although it was presented differently according to sex and city. In Talca, older adults (particularly women) were more likely to be vaccinated. This is expected, as older people are a priority group in vaccination campaigns, and because the male sample was scarce in Talca. This aligns with international studies describing higher uptake of COVID-19 vaccines in people over 45 years old [11,13].

Gender differences in vaccine uptake could partly be attributed to gender-related healthcare-seeking behaviors [49]. Chilean women tend to exhibit more proactive health-seeking behaviors [50]. Additionally, women with multiple comorbidities may have more frequent contact with healthcare centers, increasing their likelihood of vaccination. In addition, maternal care responsibilities may increase their interaction with the healthcare system, contributing to higher vaccination rates among women. A study in the United States suggests that demographic variables (age and sex) influence COVID-19 vaccination uptake more than social and economic factors [13].

In the La Serena-Coquimbo conurbation, higher education was spatially correlated with the number of vaccine doses. Health and vaccine literacy have been associated with vaccine acceptance and educational level [51,52]. COVID-19 vaccine uptake has been associated with higher economic status [11–13]. Additionally, in the conurbation’s male sample, a negative correlation was observed between the number of doses and overcrowding, reflecting lower access to vaccines in males with higher social vulnerability. This situation shows greater geographic segregation of social determinants in the conurbation compared to Talca.

Surprisingly, distance to health centers did not influence vaccination in either city, as also described in the literature [47]. This could be explained by the constant presence of health centers in the territories covering entire neighborhoods in both cities. Moreover, the low relevance of distance to health facilities confirms that structural determinants — defined here as the institutional rules, power relations, and hierarchical social arrangements that generate durable inequities among socially constructed groups [53] —still play a decisive role in shaping vaccine patterns. Within the WHO framework for social determinants of health, the health system itself operates as an intermediate determinant, mediating, but not originating, these structural forces [54].

Overall, the results show significant differences between the two cities regarding the territorial distribution of the studied variables and their relationship with COVID-19 vaccination. The differences between cities may reflect cultural factors and regional variations in attitudes toward preventive behaviors. Furthermore, it may reflect different distributions of sociodemographic factors within cities, where neighborhoods emerge that are characterized by vulnerability on the one hand and higher socioeconomic status on the other.

The research has limitations. The cross-sectional design provides only a snapshot at a given point in time, without offering insight into the evolution of disparities over longer periods. Additionally, the predominance of women in the sample may mean that the observed gender-specific disparities may not be generalizable. Socioeconomic independent variables at the census tract level (proportion elderly, educational attainment, overcrowding rate, and distance to health center) were measured at the ecological level. Therefore, this approach may introduce potential ecological fallacy, as tract-level characteristics may not reflect individual participants´ circumstances. Due to the small male sample size in Talca (n = 118), the sex-disaggregated spatial analysis may have had insufficient statistical power to detect significant spatial associations, and the absence of significant findings in this subgroup should be interpreted with caution.

Finally, as is usually the case in spatial analysis, results can only be extrapolated to cities with similar characteristics to those studied. However, it still provides information for designing vaccination campaigns or programs using a territory-based approach in cities with similar characteristics to those studied, a situation shared by cities in Chile and other Latin American countries. The minimum set of variables to consider is the population’s age, sex, and socioeconomic distribution to ensure greater equity in access to vaccination.

All the above suggest that future research should contemplate, for example, longitudinal studies to understand the dynamics of vaccination processes and their determinants, qualitative research to complement the geospatial analysis, comparative studies in different contexts or countries to make the results more generalizable, or studies to evaluate interventions or the impact of specific vaccination policies.

Recommendations arising from the results are: first, implementing geospatial surveillance systems integrated with demographic and social data sources. Second, vaccination strategies should be tailored to local contexts, accounting for demographic (sex, age) and social variables (vulnerability, educational level). Third, long-term strategies to improve health literacy and build trust in health institutions are needed to enhance access to vaccination programs. Fourth, transition plans from emergency mass vaccination to routine immunization programs should be developed proactively to anticipate and address the re-emergence of disparities.

## Conclusions

The hypothesis that there is a heterogeneous geospatial distribution of vaccination associated with socio-demographic factors after the end of the epidemic emergency is confirmed.

The study provides useful insights for policymakers and public health officials who are responsible for designing more effective and equitable public health strategies worldwide. It demonstrates the complex transition from emergency vaccination to epidemic control to a long-term sustainable response through vaccination programs. Such a transition requires proactive, adaptive, and data-driven strategies to achieve equitable immunization. Consequently, vaccination strategies need to be adapted using a territorial approach, considering the complex relationships among socio-demographic factors and unique local contexts.

## Supporting information

S1 Appendix Statistical analysis details and formulas. (PDF)

S1 Fig Maps of Talca city showing the distribution of vaccine dose clustering in women and its associations with socio-demographic factors (age over 65, educational level, overcrowding, and distance to health center). Regional boundaries and road networks were obtained from the Chilean National Statistics Institute (Instituto Nacional de Estadísticas, INE Chile) open geodata repository (https://www.ine.gob.cl/herramientas/portal-de-mapas/geodatos-abiertos. CC-BY 4.0 license: https://www.ine.gob.cl/terminos-de-uso-y-licencia-de-datos-abiertos.
(TIF)

S2 Fig Map of Talca city showing the distribution of vaccine dose clustering in men and its associations with socio-demographic factors (age over 65, educational level, overcrowding, and distance to health center). Regional boundaries and road networks were obtained from the Chilean National Statistics Institute (Instituto Nacional de Estadísticas, INE Chile) open geodata repository (https://www.ine.gob.cl/herramientas/portal-de-mapas/geodatos-abiertos. CC-BY 4.0 license: https://www.ine.gob.cl/terminos-de-uso-y-licencia-de-datos-abiertos.
(TIF)

S3 Fig Map of the La Serena/Coquimbo conurbation showing the distribution of vaccine dose clustering in women and its associations with socio-demographic factors (age over 65, educational level, overcrowding, and distance to health center). Regional boundaries and road networks were obtained from the Chilean National Statistics Institute (Instituto Nacional de Estadísticas, INE Chile) open geodata repository (https://www.ine.gob.cl/herramientas/portal-de-mapas/geodatos-abiertos. CC-BY 4.0 license: https://www.ine.gob.cl/terminos-de-uso-y-licencia-de-datos-abiertos.
(TIF)

S4 Fig Map of La Serena/Coquimbo conurbation showing the distribution of vaccine dose clustering in men and its associations with socio-demographic factors (age over 65, educational level, overcrowding, and distance to health center). Regional boundaries and road networks were obtained from the Chilean National Statistics Institute (Instituto Nacional de Estadísticas, INE Chile) open geodata repository (https://www.ine.gob.cl/herramientas/portal-de-mapas/geodatos-abiertos. CC-BY 4.0 license: https://www.ine.gob.cl/terminos-de-uso-y-licencia-de-datos-abiertos.
(TIF)
