# Supplementary material for: Geospatial disparities in post-pandemic SARS-CoV-2 vaccination: Evidence from Chile beyond the national success
Source: PLOS Glob Public Health. 2026 Jul 27;6(7):e0006808. doi: 10.1371/journal.pgph.0006808 (PMC13405313; doi:10.1371/journal.pgph.0006808)
Supplement: S1 Appendix — (PDF) [file pgph.0006808.s001.pdf]

Paper Title: Geospatial disparities in post-pandemic SARS-CoV-2 vaccination: Evidence from Chile beyond the national success

S1 Appendix. Statistical analysis details and formulas

The spatial autocorrelation index (Anselin, 1996) stems from Moran's (1948) concept of spatial autocorrelation, which is based on the premise of spatial randomness—the assumption that observations or records are spatially random. However, it is precisely this spatial randomness that serves as the null hypothesis to be rejected, since, unlike classical statistics, where phenomena exhibit randomness, spatial statistics recognizes the existence of certain types or degrees of patterns (Anselin, 1996; Anselin and Li, 2020).

It is from the rejection of this idea of spatial randomness that spatial autocorrelation arises, understood as the degree to which a geographic variable is correlated with itself at different locations within the same geographic space; that is, how values (high or low) tend to organize themselves and be located near their counterparts (Siabato and Guzmán-Manrique, 2019). In line with the above, Anselin and Li (2020) argue that spatial autocorrelation demonstrates not only how similar the values are to one another but also locational similarity, understood as the proximity between observations.

For this research, Moran's autocorrelation index (1948) was used, which is expressed by the equation:

$$I = \frac{\sum_{i=1}^{n} \quad \sum_{j=1}^{n} \quad w_{ij} z_i * \ z_j / S_0}{\sum_{i=1}^{n} \quad z_i^2 / n}$$

Where *i* corresponds to the spatial location of the observation of the variable x, denoted as *zi*, which is equal to *xi* – *μ*, where *μ* is the mean of *x*. Meanwhile, *wij* corresponds to the values of the spatial weight matrix.

As a result, we obtain an index that ranges from -1 to 1, indicating the degree of randomness or clustering of the data in space. Negative values indicate that the data do not tend to cluster in space; that is, high values are not near other high values and are grouped with low values, which is why we speak of spatially significant dispersion. Conversely, if the values in the database tend to cluster spatially, that is, high values cluster near other high values and low values cluster near other low values—we speak of a spatially significant concentration, and Moran's I will be positive, close to 1. Meanwhile, Moran's I values close to zero indicate that the data are uniformly distributed in space.

In addition, the p-value is estimated under the null hypothesis of spatial randomness (Moran's autocorrelation index equals 0), allowing us to reject or fail to reject the null hypothesis.

Figure x: explanatory diagram of the spatial autocorrelation index

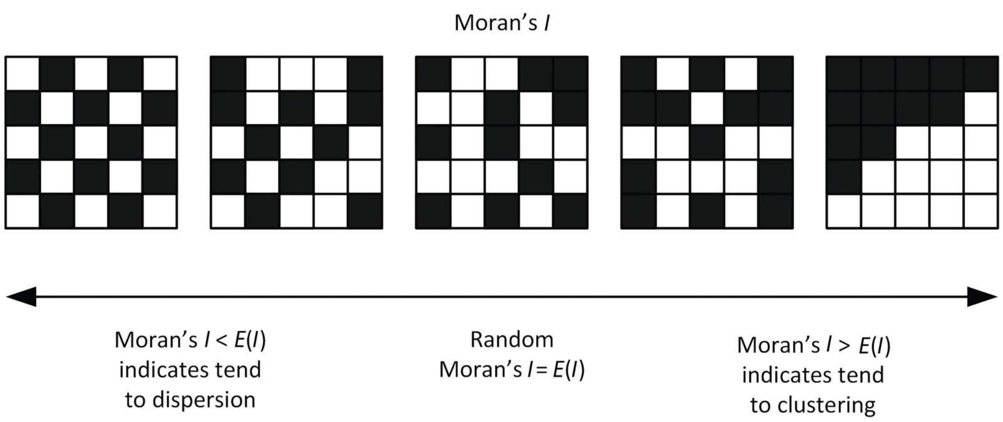

Source: Anselin, 2024.

Based on the value of the spatial autocorrelation index, using the spatial weight matrix derived from the locations of the observations, we can define the local index of spatial autocorrelation (LISA), as defined by Anselin (1995), as the ability to identify the formation of clusters (statistically significant spatial concentrations of high or low values) in space, as well as the existence of outliers.

In mathematical terms, the local index spatial autocorrelation is expressed as:

$$LISA = C * z_i \sum_{j=1}^{n} w_{ij} z_i$$

Where C corresponds to an adjustment of the denominator in the global autocorrelation index equation, based on which the value i for a location is estimated as a function of its spatial lag—defined as the weighted sum of the values of nearby locations.

These clusters are defined as High-High clusters and Low-Low spatial clusters, corresponding to spatial concentrations of high values (HH) or low values (LL) that are statistically significant in space; while High-Low spatial outliers and Low-High spatial outliers are also identified, corresponding to outliers—that is, high values (HL) in areas where low values are concentrated, or low values (LH) in areas where high values are concentrated.

The interpretation of the LISA index is the same as that described above for spatial autocorrelation.

References

1. Anselin, L. (1995). Local indicators of spatial association – LISA. Geogr Anal, 1995, 27(2):93–115
2. Anselin, L. (1996). The Moran Scatterplot as an ESDA Tool to Assess Local Instability in Spatial Association. In Spatial Analytical Perspectives on GIS in Environmental and Socio-Economic Sciences, edited by Manfred Fischer, Henk Scholten, and David Unwin, 111–25. London: Taylor; Francis.
3. Anselin, L. & Li, X. (2020) Tobler's Law in a Multivariate World." Geographical Analysis. 52: 494–510. https://doi.org/10.111/gean.12237.
4. Moran, P. (1948). "The Interpretation of Statistical Maps." Journal of the Royal Statistical Society, B 10: 243–51.
5. Siabato, W. & Guzmán-Manrique, J. (2019). La autocorrelación espacial y el desarrollo de la geografía cuantitativa. Cuadernos de Geografía: Revista Colombiana de Geografía. 28 (1): 1-22. https://doi.org/10.15446/rcdg.v28n1.76919
