# Supplementary material for: Geospatial disparities in post-pandemic SARS-CoV-2 vaccination: Evidence from Chile beyond the national success
Source: PLOS Glob Public Health. 2026 Jul 27;6(7):e0006808. doi: 10.1371/journal.pgph.0006808 (PMC13405313; doi:10.1371/journal.pgph.0006808)
Supplement: Attachment — Submitted filename: Point-by-point response2-2.pdf [file pgph.0006808.s006.pdf]

Editor comments:

While the Methods are adequate for conducting a population-based seroprevalence survey (as published previously by some of the co-authors), the number of SARS-CoV-2 vaccine doses can be very poorly captured by participant report. It is therefore not a rigorous enough approach to data collection for this key variable to accept the conclusions presented (4 in PLOS GPH Criteria for Publication).

Answers:

Thank you for this important methodological concern. We acknowledge that our original manuscript did not adequately describe our comprehensive approach to vaccination data collection, which may have created the impression that we relied on participants self-report.

Our Study Data Collection Strategy:

We implemented a comprehensive two-tier approach specifically designed to address the limitation of self-reported vaccination data:

1. **Primary method- Objective documentation:** Participants were asked to either access their online vaccination records through the national immunization registry portal or present their physical vaccination cards.
2. **Secondary method-Direct Registry Access**: When the primary method was not feasible, participants could sign an authorization permitting the research team to directly request their COVID-19 vaccination records from the national immunization program.

Ethical Approval:

We obtained formal approval (Amendment 30-2023-E2) from our Institutional Review Board specifically authorizing direct access to vaccination records for participants without physical vaccination cards, ensuring full compliance with research ethics standards. We have included the amendment and authorization documents in both original language and translation

Supporting Infrastructure in Chile:

This methodology was feasible due to Chile's robust digital health infrastructure. According to the most recent official data from the Telecommunications Regulatory Authority (SUBTEL), 96.5% of Chilean households report having paid internet access, with smartphone usage reaching 94.8% of the population daily (page 16) (SUBTEL, 2024). However, important demographic variations exist, while smartphone use

approaches universality among younger adults (98.8% in 16-44 age groups), it declines to 84.0% among adults aged 60 and older, reflecting a notable digital divide in this population (page 42).

This systematic approach eliminates reliance on participant recall for vaccination status, instead utilizing official government records as the primary data source.

We will incorporate this detailed methodology description in our manuscript revision to ensure complete transparency regarding our rigorous data collection procedures and address any concerns about the reliability of our vaccination data.

Reference: SUBTEL. Final Report "Eleventh Survey on Internet Access, Uses, and Users in Chile." November 16, 2024. Available at: https://www.subtel.gob.cl/wp-content/uploads/2025/02/Informe-Final-Subtel-Acceso-y-Uso-Internet-2024.pdf

In addition, following submission requirements check by the editorial team, we have updated the legends of Figures 1–3 and the supporting maps to include explicit attribution of all cartographic base layers. Regional and county administrative boundaries and road networks were sourced from the open geodata repository of the Chilean National Statistics Institute (INE Chile; https://www.ine.gob.cl/herramientas/portal-de-mapas/geodatos-abiertos), under terms compatible with CC BY 4.0. Attribution is also displayed in the lower right corner of each map. No proprietary mapping services were used.
