# Supplementary material for: Geospatial disparities in post-pandemic SARS-CoV-2 vaccination: Evidence from Chile beyond the national success
Source: PLOS Glob Public Health. 2026 Jul 27;6(7):e0006808. doi: 10.1371/journal.pgph.0006808 (PMC13405313; doi:10.1371/journal.pgph.0006808)
Supplement: Attachment — Submitted filename: Point-by-point response.pdf [file pgph.0006808.s007.pdf]

**Point-by-point answer to reviewers**

PGPH-D-25-02096R1

Geospatial disparities in post-pandemic SARS-CoV-2 vaccination: Evidence from Chile beyond the national success

Thanks for the careful revision of our manuscript. We will address all the comments the reviewers made.

**Editor comment:**

While the Methods are adequate for conducting a population-based seroprevalence survey (as published previously by some of the co-authors), the number of SARS-CoV-2 vaccine doses can be very poorly captured by participant report. It is therefore not a rigorous enough approach to data collection for this key variable to accept the conclusions presented.

**Response**

Chile is among the ten OECD countries with the best digital government. Among the three countries, Chile recorded the most pronounced increase in score in the 2025 Governance Digital Government Index (1), reflecting a substantial commitment to advancing digital governance policies.

Chile has maintained a National Immunization Program (PNI, Programa Nacional de Inmunizaciones) since 1978, which has substantially reduced morbidity and mortality from vaccine-preventable diseases and contributed to a marked decline in infant mortality. Among its most notable achievements are the eradication of smallpox (1950), the elimination of polio (1975), and measles (1992) (2). The National Immunization Program has a comprehensive approach and aims to prevent morbidity, disability, and death from vaccine-preventable diseases throughout the entire life cycle. (2)

Chile's leadership in preventing communicable diseases extends beyond its longstanding immunization infrastructure. In 2023, Chile became the first country in the southern hemisphere to implement a universal immunization strategy with nirsevimab — a monoclonal antibody for the prevention of respiratory syncytial virus (RSV) — demonstrating once again its capacity for rapid and comprehensive public health innovation, which was highlighted by The Lancet on the cover of its November 2025 issue (3). A national evaluation of this strategy, covering 154,173 infants during the 2024 RSV season, demonstrated a combined effectiveness of 76.4% against RSV-related lower respiratory tract infection hospitalizations and 84.9% against RSV-related intensive care unit admissions, and no deaths among children under one year of age in the last two winters. These findings, alongside an estimated 77.5% relative reduction in RSV-related hospitalizations, underscore Chile's ability to translate advanced digital health infrastructure — including the RNI and PNI — into measurable reductions in the burden of communicable diseases at the population level (3).

**National Immunization Registry**

The National Immunization Registry (Registro Nacional de Inmunizaciones, RNI) was established during the 2010 H1N1 influenza pandemic. In 2011, it began registering the routine vaccines administered under the National Immunization Program. By 2013, the National Immunization Registry had become the official information source for monitoring vaccine coverage within Chile's national vaccination schedule (2). The National Immunization Registry is a digital platform that provides the Ministry of Health (Ministerio de Salud, MINSAL) with an

individualized electronic record of all vaccinations administered nationwide, ensuring full traceability of each biological product and vaccine recipient across both public and private vaccination centers operating under agreements with the national health authority (2).

The National Immunization Program vaccines (4) are mandatory and free for all target population groups residing in the country (5), regardless of their health insurance coverage. Chile's health system comprises two schemes: the public system (FONASA), which covers 88% of the population in La Serena/Coquimbo and 98% in Talca (6, 7), and the private system (ISAPRE) (8).

The PNI also administered a public-facing digital platform (Patient Portal: https://portalpaciente.minsal.cl/portal) through which individuals can access their personal vaccination history and obtain information on recommended vaccines. This tool complements the RNI by providing patients with direct access to their immunization records, thereby promoting transparency and vaccine uptake.

The following screenshot of the [Portal Paciente] interface displays the vaccination information accessible to registered users.

<table>
<tr><td>Patient platform<br>https://portalpaciente.minsal.cl/portal<br>The web was translated using Google Translate</td><td>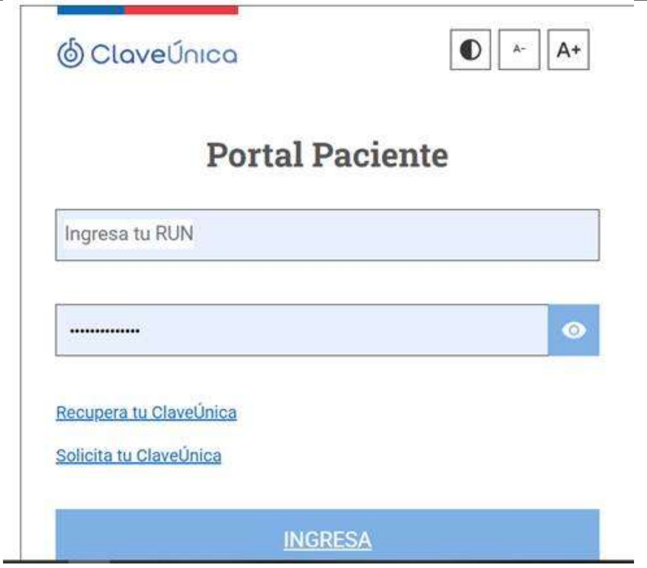
</td></tr>
<tr><td>Greeting of the patient</td><td>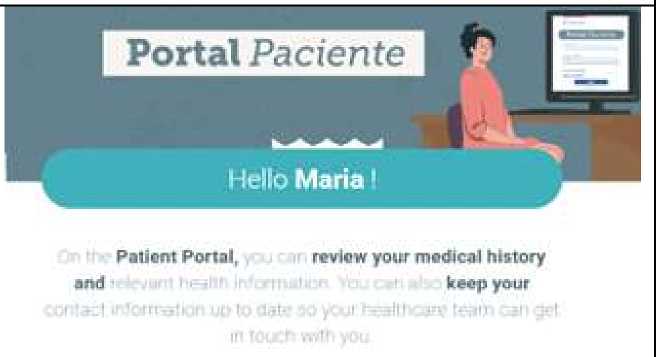
</td></tr>
</table>

<table>
<tr><td>Available information:</td><td>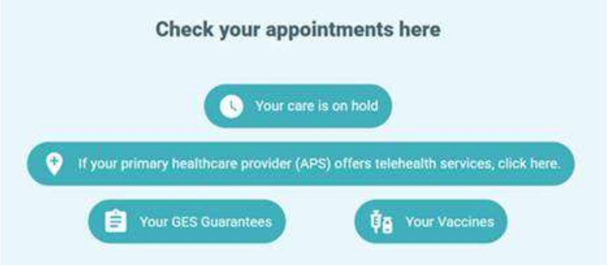
</td></tr>
<tr><td colspan="2">Selecting “your vaccine” allows users to retrieve detailed information, including the vaccine name, date of administration, and dose number when applicable.</td></tr>
</table>

In our study, we obtained 25 vaccine records through the Patient Portal; all other records were obtained from the National Immunization Registry. This information was incorporated to result in Section Line 189-191.

**References**

1. OECD Working Papers on Public Governance Digital Government Index and Open, Useful and Re-usable Data Index 2025 Results and Key Findings No. 90. Available from: chrome-extension://efaidnbmnnnibpcajpcglclefindmkaj/https://www.oecd.org/content/dam/oecd/en/publications/reports/2026/02/digital-government-index-and-open-useful-and-re-usable-data-index_dbe102ed/6347ec74-en.pdf
2. Registro Nacional de Inmunizaciones (RNI). Available from: https://vacunas.minsal.cl/registro-nacional-de-inmunizaciones/#preguntasfrecuentes
3. Torres JP, Sauré D, Goic M, Thraves C, Pacheco J, Burgos J, Trigo N, Del Solar F, Neira I, Díaz G, O'ryan M. Effectiveness and impact of nirsevimab in Chile during the first season of a national immunisation strategy against RSV (NIRSE-CL): a retrospective observational study. The Lancet Infectious Diseases. 2025 Nov 1;25(11):1189-98
4. Vacunas del Programa Nacional de Inmunizaciones del  Minsal. Available from: https://www.chileatiende.gob.cl/fichas/105622-vacunas-del-programa-nacional-de-inmunizaciones-del-minsal
5. CHILE.  Ministerio de Salud. (25 de septiembre de 2021). Decreto 50 Exento. Dispone Vacunación Obligatoria contra Enfermedades Inmunoprevenibles de la Población del País. [Consultado el 28 de mayo de 2026] Disponible en: https://bcn.cl/2ruro
6. SIIT Estadísticas Territoriales. Series estadísticas oficiales a nivel comunal y regional, provenientes de organismos del estado en ambitos demográficos, educacionales, económicos, salud, sociales y seguridad ciudadana. Biblioteca del Congreso Nacional de Chile. Estadisticas territoriales. Disponible en: Disponible en: https://www.bcn.cl/siit/estadisticasterritoriales/resultados-consulta?id=542303
7. Reporte Comunal | 2024. Biblioteca del Congreso Nacional de Chile. Disponible en: https://www.bcn.cl/siit/reportescomunales/comunas_v.html?anno=2024&idcom=7101
8. Mella PF, Narváez CG. Análisis del sistema de salud chileno y su estructura en la participación social. Saúde em Debate. 2023 Feb 27;46:94-106. https://doi.org/10.1590/0103-11042022E407

| Reviewer Comments | Response |
| --- | --- |
| **Reviewer #1:** | |
| This manuscript explores the geospatial disparities in post-pandemic SARS-CoV-2 vaccination in Chile, providing valuable insights that can be used in public health control of infectious diseases.<br><br>The proposed framework has potential applicability to research on understanding health behavior in relation to other regions and other diseases, particularly emerging and re-emerging infections.<br><br>The authors deserve appreciation for the work that they put into designing the experiments and collecting the data using proper channels and in compliance with ethical considerations. | Thank you for your appreciation of our work and for recognizing its value. We appreciate all suggestions for improving readability. |
| Certain claims could benefit from references to existing literature, thus lending more credibility to the research process (e.g., Line 56, Line 58, Line 90, Line 103, Line 126). | References have been added to all lines suggested. Please note that line numbers have shifted throughout the manuscript due to subsequent revisions. Line 56, 58, 95,120, 136 |
| Please describe the variables used in relation to the categories that they belong to. For example, whether they are discrete or continuous. | We incorporated a table containing information on the variables: description, type of variable, and data source.<br>Line 148 |
| More information regarding the statistical test (including any formulas) should be provided under the dedicated section “Statistical analysis”. Spatial autocorrelation analysis needs to be defined properly with sufficient information. All statistical methods used should be described in this section, including Moran’s Index, Expected index, Z score, and P Value with reference to the context of the study. | Supplementary material with more detailed information on the geospatial analysis is provided. S1 Appendix. Statistical analysis details and formulas. |
| The manuscript seems to lack graphical illustrations; while not necessary, it would be better to include a few figures effectively communicating the statistical results. | Three figures are included in the main manuscript, with additional figures provided as supporting information. All materials were uploaded to the journal submission platform. |
| While not necessary, the authors could discuss how the proposed framework could be adapted to other regions or international contexts as part of | The points raised by the reviewer are addressed in the last two paragraphs of the discussion.<br>Lines 355-362 |

| | |
|---|---|
| future research. | |
| It is better to number the sections on the manuscript to improve its readability and navigation. | We have added subheadings to the Methodology and Results sections to improve readability and navigation. |
| Please carefully review the specific points below and revise the manuscript accordingly. | |
| Lines 60-64: Is employment or profession not a considerable factor? | Employment has been incorporated as a variable in the revised manuscript (Line 64). |
| Lines 71-72: Better to restructure this sentence in the past tense. | The sentence at Lines 71–72 has been restructured in the past tense as suggested. |
| Line 80: Missing reference. | Reference was incorporated. Line 80 |
| Line 82: Please revise as “specific population groups who are at more risk”. | The suggestion was incorporated. Line 82: |
| Line 83: Change “good coverage” to “sufficient vaccination coverage”? | The suggestion was incorporated. Line 83 |
| Line 91: Change “geospatial vaccination distribution post-pandemic SARS-CoV-2 vaccination” to “geospatial distribution of post-pandemic SARS-CoV-2 vaccination”? | The suggestion has been incorporated at Line 96 |
| Lines 94-95: Please rephrase for clarity. | We rephrased for clarity, Lines 98-100<br>The underlying hypothesis is that, once the epidemic ended, socio-demographic factors shaped access to vaccination, resulting in a heterogeneous geographical distribution of immunization. |
| Line 103: Change to past tense? | The phrase has been changed to past tense at Line 106. |
| Line 114 and more: Links may not be appropriate on the main text. Please check with the journal instructions. | The journal required adding a reference for the map boundary shapefile |
| Line 144: Is the phrase “than expected” necessary? | We removed "expected." Line 168 |

| | |
|---|---|
| Line 179: Change “by city” to “in terms of the city”? | The suggestion has been incorporated at Line 198 |
| Table 1: Change “No vaccinated” to “No vaccination” or “Unvaccinated”? | The suggestion has been incorporated. Now it is Table 2. |
| Line 187: Change “masculine” to “men’s” or “male”? | Masculine has been changed to 'male' at Line 209 |
| Line 226: Change “in both” to “across both”? | The suggestion has been incorporated at Line 246 |
| Line 240: Isn't the heterogeneity itself a finding of the study? So, how about phrasing the sentence as follows? "The findings show that there is some heterogeneity in the geospatial distribution of vaccination, while also confirming the hypothesis that this observation is related to socio-demographic factors in both cities." | The suggestion has been incorporated at lines 258-260 |
| Lines 245-246: Would it be possible to plot a figure to illustrate this result? | We do not include a map because vulnerability was not one of the variables analyzed; rather, it reflects the research team’s understanding of the cities. |
| Line 248: Are the correlations strong enough to warrant the use of the word “precisely”? If not, it would be better to use a different word such as "generally". | “Precisely” has been changed to “generally” at Line 267. |
| Line 282: Is the correlation with the number of doses really negative? | Yes, the correlation is indeed negative. As shown in Table 3 (La Serena–Coquimbo), Moran's I value is −0.144, confirming a negative spatial correlation with the number of doses and overcrowding.<br><br>The supplemental material on statistical analysis provides more detail in the analysis. |
| Lines 286-287: Change “as described” to “as also described”? | The suggestion has been incorporated at Line 320 |
| Line 290: Use a more appropriate adjective instead of “deeper”? | We have replaced “deeper” for “complex, multidimensional” at Line  324 |

| | |
|---|---|
| Line 315: Change “develop” to “developing”? | We have revised the whole paragraph to improve grammatical consistency and readability.<br><br>Line 358-362 |
| Line 319: Change “re-emerge” to “re-emergence”? | The suggestion has been incorporated at Line 362 |
| **Reviewer #2:** | |
| **Major Comments** | |
| The topic is timely and relevant, and the Chilean context provides a valuable comparative lens given the country’s initially equitable vaccination performance during the pandemic. The study addresses an important gap in understanding post-pandemic vaccination dynamics and offers useful insights into emerging socio-spatial disparities. | Thank you for your appreciation of our work and for recognizing its value. |
| The "WHO recommendations (reference)" placeholder<br><br>In the Introduction (line 80), the sentence reads: "Following the WHO recommendations (reference), since 2023 the Chilean Ministry of Health…" The citation marked "(reference)" remains unfilled. This should be corrected before publication. | The reference was incorporated into the text. |
| 2. Disconnect between individual-level data and census-level independent variables<br><br>The study collects individual-level data on vaccination (number of doses per person), then assigns socio-demographic independent variables at the census tract level (proportion elderly, educational attainment, overcrowding rate, distance to health center). This is a form of ecological analysis applied to individual-level outcomes, which creates the potential for an ecological fallacy tract-level characteristics may not reflect the individual participant's circumstances. This is not acknowledged in the limitations section, and readers should be cautioned against inferring individual-level causal relationships from these associations. The | The following sentence was added to the limitation section at Lines 336-343<br><br>Socioeconomic independent variables at the census tract level (proportion elderly, educational attainment, overcrowding rate, and distance to health center) were measured at the ecological level. Therefore, this approach may introduce potential ecological fallacy, as tract-level characteristics may not accurately reflect individual participants´ circumstances. |

| | |
|---|---|
| authors should either address this limitation or, if they consider their analytical approach to sufficiently mitigate it, explain why. | |
| 3. Inconsistency in the "Conclusion" column of Tables 2 and 3<br><br>There are inconsistencies in the interpretation of spatial autocorrelation results. Several variables with statistically significant p-values ($p < 0.05$) are described as having "no spatial association," despite evidence of weak but significant spatial autocorrelation based on Moran's I. The manuscript would benefit from a clearer distinction between statistical significance (p-value) and strength of association (magnitude of Moran's I). Even small Moran's I values, when statistically significant, indicate non-random spatial structure and should be interpreted accordingly (e.g., "weak but significant spatial association"). | We have included a methodological appendix (**S1 Appendix.** Statistical analysis details and formulas)<br>that explains in greater detail how to interpret Moran's I, specifying that:<br><br>*As a result, we obtain an index that ranges from -1 to 1, indicating the degree of randomness or clustering of the data in space. Negative values indicate that the data do not tend to cluster in space; that is, high values are not near other high values and are grouped with low values, which is why we speak of spatially significant dispersion (Figure X). Conversely, if the values in the database tend to cluster spatially, that is, high values cluster near other high values and low values cluster near other low values—we speak of a spatially significant concentration, and Moran's I will be positive, close to 1. Meanwhile, Moran's I values close to zero indicate that the data are distributed completely randomly in space.* |
| 4. Interpretation of age-related findings in La Serena/Coquimbo<br><br>The interpretation of the age-related findings for La Serena/Coquimbo requires clarification. The results indicate that the proportion of older adults is not spatially associated with vaccination in the total sample or among women, but is associated among men (Table 3; Results section around lines 202–209).<br><br>However, the Discussion suggests that clusters of adults younger than 65 receiving more vaccine doses may reflect workplace vaccination access (lines 267–269). This explanation appears speculative, as the study does not include occupational data, workplace vaccination records, or variables capturing access through employment.<br><br>This interpretation should either be removed or clearly framed as a hypothesis requiring further | We removed this sentence. |

| | |
|---|---|
| investigation. The manuscript should avoid attributing observed spatial patterns to mechanisms not supported by the available data. | |
| 5. Sample size and sex-disaggregated analysis<br><br>The male sub-samples particularly for Talca (n = 118) are modest. The authors acknowledge the male sample was "scarce in Talca" (Discussion, line 265) but do not address the statistical implications for the sex-disaggregated spatial analyses. With a small and spatially distributed male sample, the absence of significant spatial associations in the Talca male sub-sample may reflect limited statistical power rather than a true absence of spatial patterning. This should be discussed explicitly as a limitation rather than treated symmetrically with the female and total sample results. | We added this paragraph to the limitations section. Line 340-343<br>Due to the small male sample size in Talca (n = 118), the sex-disaggregated spatial analysis may have had insufficient statistical power to detect significant spatial associations, and the absence of significant findings in this subgroup should be interpreted with caution. |
| 6. Two-tier vaccination verification: incomplete manuscript integration<br><br>The manuscript describes a two-tier approach for verifying vaccination status (participant-provided documentation and registry confirmation with authorization; lines 129-135). While this strengthens confidence in the data collection process, the reporting remains largely procedural.<br><br>The manuscript does not provide a quantitative breakdown of how many participants were verified through each method, nor does it report any instances of missing, unverifiable, or incomplete records.<br><br>Given that vaccination status is the primary outcome, greater transparency on data verification and completeness would strengthen the credibility of the findings. A concise quantitative summary of the verification process should be included in the manuscript, rather than only in the response letter. | In the results section, we added the proportion of participants verified through each method. Lines 193-195 |
| Minor Comments | |
| Line 80 (Introduction): The phrase "vaccination coverage is also deteriorating" (present tense) in a description of trends | The suggestion has been incorporated at Line 89 |

| | |
|---|---|
| since 2023 is grammatically inconsistent with the surrounding paragraph. Suggest: "vaccination coverage has also been deteriorating." | |
| Table 1: The total for Talca is listed as n = 336 in the table header, but n = 335 in the Results text (line 161). This discrepancy should be resolved. | The total population in Talca is 336. We corrected the total number in the table; it is now Table 2. |
| Lines 315-316 (Recommendations): The sentence "Third, develop long-term strategies to improve health literacy and trust-building initiatives may improve the access to vaccination programs" is grammatically incomplete. This may be revised as follows " Third, long-term strategies to improve health literacy and build trust may enhance access to vaccination programs." | We have revised the whole paragraph to improve grammatical consistency and readability.<br><br>Line 355-362 |
| Reference 9 is an exact duplicate of Reference 5 (Tesfa GA et al., PLoS One 2023, doi:10.1371/journal.pone.0279399). One should be removed or replaced with the intended citation. | We have removed the duplicate reference. |
| Conclusion and recommendation<br><br>Major revision required. The manuscript addresses an important and underexplored topic, and the Chilean context provides a compelling natural experiment for studying the post-pandemic re-emergence of socio-demographic determinants of vaccination. However, the comments outlined above need to be resolved before the paper meets PLOS Global Public Health's publication criteria. | We thank the reviewers for their thorough and constructive evaluation. We believe that the revisions made in response to all comments have substantially strengthened the manuscript and addressed the key methodological and interpretive concerns raised. We trust that the revised version now meets the publication criteria of PLOS Global Public Health. |
| **Reviewer #3:** | |
| Overall: The topic and geospatial approach are relevant and important. However, the authors have not sufficiently justified the approach and some of the objectives are not | We thank the reviewers for recognizing the value of our work. We have addressed all comments and believe the manuscript has been substantially improved. |

| | |
|---|---|
| clear. | |
| lines 61-64: The fragmentation of the referencing here affects the flow of the sentence and can be improved by regrouping the factors with same references so they can be cited together. e.g. age and insurance status [14, 18]. instead of age [14.18], and insurance status [18]. | We believe that readers may want to know the exact references for each factor influencing vaccination uptake; however, we have collapsed the references for age and insurance status [14, 18] as suggested. Line 64 |
| Line 80: No reference included for the WHO recommendation | The reference was added to the manuscript. Line 80 |
| lines 83-85: This information is important, as it describes post-pandemic vaccine hesitancy, however, I expect also to see why vaccination is still expected or seen to be important in Chile in 2024. | We added this explanation. Line 83-87<br>Vaccines against SARS-CoV-2 were still necessary for individuals at risk of serious disease in 2024. The elderly and those with chronic conditions remain particularly vulnerable to severe outcomes. Additionally, the emergence of new variants and vaccine escape mechanisms justifies the continuation of vaccination campaigns, in line with the recommendations of Chilean health authorities. |
| line 91/table 1 and across the paper: It is not clear in this paper what represents the post-pandemic period. This is also important in understanding the vaccine doses reported because some of the doses may have been taken in the pandemic period. | The WHO declared the end of the COVID-19 pandemic in May 2023, while Chile declared the end of the pandemic on 31 August 2023. This information has been added to the introduction. Line 71<br><br>https://www.gob.cl/noticias/fin-alerta-sanitaria-covid-19-coronavirus-enfermedades-respiratorias-mascarillas-teletrabajo/ |
| 92-93:justification for choosing these cities. | An explanation has been incorporated in the Methods section. Line 109-111 |
| 105-108: how were the samples collected, transported and stored? what type of test was done. | Blood samples were collected from participants, and serum was obtained to measure IgG antibodies against SARS-CoV-2 using an enzyme-linked immunosorbent assay (ELISA). Serum samples were stored at −20°C until analysis.<br>This information was not included in the manuscript, as the study focused on vaccines received by participants. |
| 130-135: how reliable are these different | The capabilities of the Chilean system are |

| | |
|---|---|
| sources of vaccination records and how do they compare | described above. |
| The analysis by sex is good. However, the background and objectives did not give any indication of importance or interest in the disparities by sex. | We have added sex-specific information regarding the vaccine in three sections: the Introduction (line 64), the objective (line 98), and the Methodology (lines 160–162). |
| Linea 155-156: what is the reference that informs this cut off for overcrowding? | In Chile, the Ministerio de Desarrollo Social y la Familia (MIDESO, 2024) in collaboration with the Instituto Nacional de Estadísticas (INE, 2024), measures overcrowding through the CASEN National Survey, defining it as a situation in which an average of 2.5 people live in each bedroom used exclusively for sleeping within a household.<br><br>Line 146<br><br>**References added to the manuscript. Line 146**<br>Ministerio de Desarrollo Social y Familia. (2024). Resultados Encuesta Casen 2024. Gobierno de Chile. https://observatorio.ministeriodesarrollosocial.gob.cl/encuesta-casen-2024<br>Instituto Nacional de Estadísticas (INE). (2024).<br><br>Censo de Población y Vivienda 2024: Síntesis de resultados. Ministerio de Economía, Fomento y Turismo, Gobierno de Chile. https://censo2024.ine.gob.cl/wp-content/uploads/2025/12/sintesis_resultados_censo2024.pdf |
| Table 1: What vaccines constitutes the basic scheme? Please specify the bivalent types or explain briefly in a footnote. | We have incorporated two footnotes into Table 2 (previously Table 1).<br>Line 200-202 |
| Line 180 (table 1) and other tables: This may not be necessary. Stating sources is more important if the source is from a different study or secondary data. | The phrase was eliminated from each table. |
| Tables 2 and 3: what determines if an association is slight spatial association? What | We have incorporated a supplemental file with a more detailed explanation of the analysis. |

| | |
|---|---|
| are the other categories of spatial associations? | |
| Lines 237-331: In this discussion, I expect a synthesis that compares and contrast with post-pandemic hesitancy in other countries particularly of the some geographic and/or socioeconomic similarities. Where possible, discussion should also highlight similar geospatial approaches from other studies on COVID-19 or other vaccines used in pandemics or epidemics | We have incorporated the following paragraph into the discussion section. Line 270-284<br><br>Systematic reviews and meta-analyses have reported COVID-19 vaccine hesitancy rates ranging from 42% (in Africa) to 16% (in Europe) (Tianshuo Zhao et al., 2025), with acceptance rates also varying by country and continent, ranging from 13% (Iraq) to 92% (Tunisia) (Malik Sallam et al., 2022). Various factors influence vaccine acceptance or hesitancy. For example, a county-level study conducted in the United States attributed 79% of the variation in vaccination rates to spatial patterns. The study shows that per capita income and the percentage of minorities have positive effects on vaccination rates, while age, mobile homes, and the uninsured population have negative effects on vaccine uptake (A. Mollalo & Moosa Tatar, 2021). A South African study using geo-additive models revealed geographically varying levels of vaccine hesitancy, with strong geospatial correlations between such hesitancy and beliefs (H. Wand et al., 2023). Several factors linked to COVID-19 vaccine hesitancy have been identified in conventional and geospatial studies (H. Wand et al., 2023; Rahbeni T et al., 2024). One of the most significant is trust—both in the vaccine itself and in governments—where health and vaccine literacy play a key role (Carrieri, V., 2023 ; Romate, J 2022; Will Jennings et al 2023; Roy, D. 2022 ; Pourrazavi S, 2023). Common to most of the studies reviewed is the recommendation to adapt vaccination strategies to local contexts. |
| **Reviewer #4:** | |
| The manuscript addresses a very important issue of public health significance - sustaining equitable vaccine coverage following pandemics. The message is clear, convincing, and backed by sufficient evidence, which is fairly well referenced. Some English language editing of the | We thank the reviewers for recognizing the value of our work. |

| | |
|---|---|
| manuscript is required | |
| **Figure Resubmissions:** | |
| While revising your submission, we strongly recommend that you use PLOS's NAAS tool (https://ngplosjournals.pagemajik.ai/artanalysis) to test your figure files. NAAS can convert your figure files to the TIFF file type and meet basic requirements (such as print size, resolution), or provide you with a report on issues that do not meet our requirements and that NAAS cannot fix.<br>After uploading your figures to PLOS's NAAS tool - https://ngplosjournals.pagemajik.ai/artanalysis, NAAS will process the files provided and display the results in the "Uploaded Files" section of the page as the processing is complete. If the uploaded figures meet our requirements (or NAAS is able to fix the files to meet our requirements), the figure will be marked as "fixed" above. If NAAS is unable to fix the files, a red "failed" label will appear above. When NAAS has confirmed that the figure files meet our requirements, please download the file via the download option, and include these NAAS processed figure files when submitting your revised manuscript. | We have used PLOS's NAAS tool to check our figures for format, resolution, and print size. |
