# Supplementary material for: Geospatial disparities in post-pandemic SARS-CoV-2 vaccination: Evidence from Chile beyond the national success
Source: PLOS Glob Public Health. 2026 Jul 27;6(7):e0006808. doi: 10.1371/journal.pgph.0006808 (PMC13405313; doi:10.1371/journal.pgph.0006808)
Supplement: Attachment — Submitted filename: Point-by-point_response_auresp_3.pdf [file pgph.0006808.s008.pdf]

PGPH-D-25-02096R2

Geospatial disparities in post-pandemic SARS-CoV-2 vaccination: Evidence from Chile beyond the national success

PLOS Global Public Health

**Editor Comments**	**Response**
I have found you responded well to reviewers. As the editor, I just have one larger question. What do you mean by "structural issues"? The word "structural," in my mind, encompasses distance to clinic - which you list as something which is not structural in page 18 line 320. Essentially, I think you need to slightly discuss structural and social determinants a bit more - or at least define them more precisely - in the discussion. I would think this is a minor change, and I will be able to process your manuscript rapidly with these edits - or a response to me if you do not think edits are warranted.	Thanks for acknowledging our good response to reviewers.<br>The WHO framework recognizes the health system as an intermediate determinant of health. In this context, the phrase is correct. Nevertheless, we have modified the paragraph to clarify this idea.<br><br>**Reference.**<br>Solar O, Irwin A. A Conceptual Framework for Action on the Social Determinants of Health. World Health Organization Commission on the Social Determinants of Health; 2010. Accessed October 13, 2022. https://www.who.int/publications-detail-redirect/9789241500852<br><br>Briefly, social determinants of health are defined as *"the conditions in which people are born, grow, live, work and age, and the wider forces shaping daily life."* On the other hand, structural determinants of health are understood to be *"the socioeconomic and political mechanisms that generate and distribute power, resources, and social position, thereby shaping the social determinants of health and producing health inequities."* The structural determinants of health are 1) the written and unwritten rules that create, maintain, or eliminate durable and hierarchical patterns of advantage among socially constructed groups in the conditions that affect health, and 2) the manifestation of power relations in that people and groups with more power based on current social structures work—

	implicitly and explicitly—to maintain their advantage by reinforcing or modifying these rules.<br><br>**Reference.**<br>Heller JC, Givens ML, Johnson SP, Kindig DA. Keeping It Political and Powerful: Defining the Structural Determinants of Health. Milbank Q. 2024 Jun;102(2):351-366. doi: 10.1111/1468-0009.12695. Epub 2024 Feb 16. PMID: 38363858; PMCID: PMC11176401.
