# Supplementary material for: Geospatial disparities in post-pandemic SARS-CoV-2 vaccination: Evidence from Chile beyond the national success
Source: PLOS Glob Public Health. 2026 Jul 27;6(7):e0006808. doi: 10.1371/journal.pgph.0006808 (PMC13405313; doi:10.1371/journal.pgph.0006808)
Supplement: Peer review history

# Editor report: Decision Letter 0

**DOI:** 10.1371/journal.pgph.0006808.r001

**Authors:** Eugene T. Richardson

16 Sep 2025

PGPH-D-25-02096

Geospatial disparities in post-pandemic SARS-CoV-2 vaccination: Evidence from Chile beyond the national success

PLOS Global Public Health

Dear Dr. Núñez-Franz,

Thank you for submitting your manuscript to PLOS Global Public Health. After careful consideration, we have decided that your manuscript does not meet our criteria for publication and must therefore be rejected.

Specifically:

==============================

While the Methods are adequate for conducting a population-based seroprevalence survey (as published previously by some of the co-authors), the number of SARS-CoV-2 vaccine doses can be very poorly captured by participant report. It is therefore not a rigorous enough approach to data collection for this key variable to accept the conclusions presented (#4 in PLOS GPH Criteria for Publication).

==============================

We are sorry that we cannot be more positive on this occasion. We very much appreciate your wish to present your work in one of PLOS's Open Access publications. Thank you for your support, and we hope that you will consider PLOS Global Public Health for other submissions in the future.

Yours sincerely,

Eugene T. Richardson, MD, PhD

Academic Editor

PLOS Global Public Health

Additional Editor Comments (if provided):

[Note: HTML markup is below. Please do not edit.]

Reviewers' comments:

[NOTE: If reviewer comments were submitted as an attachment file, they will be attached to this email and accessible via the submission site. Please log into your account, locate the manuscript record, and check for the action link "View Attachments". If this link does not appear, there are no attachment files.]

# Author comment: Author response to Decision Letter 1

**DOI:** 10.1371/journal.pgph.0006808.r002

31 Mar 2026

Attachment Submitted filename: Point-by-point response2-2.pdf

# Peer review: Decision Letter 1

**DOI:** 10.1371/journal.pgph.0006808.r003

**Authors:** Abram L. Wagner

5 May 2026

PGPH-D-25-02096R1

Geospatial disparities in post-pandemic SARS-CoV-2 vaccination: Evidence from Chile beyond the national success

PLOS Global Public Health

Dear Dr. Núñez-Franz,

Thank you for submitting your manuscript to PLOS Global Public Health. After careful consideration, we feel that it has merit but does not fully meet PLOS Global Public Health’s publication criteria as it currently stands. Therefore, we invite you to submit a revised version of the manuscript that addresses the points raised during the review process.

Please respond to reviewer comments, but let us know in your response if you do not feel the need to respond to a particular comment. Please also be aware, that as I review your response, I'll also keep in mind the previous editors' comment - addressing that in your response, too, would be helpful:

While the Methods are adequate for conducting a population-based seroprevalence survey (as published previously by some of the co-authors), the number of SARS-CoV-2 vaccine doses can be very poorly captured by participant report. It is therefore not a rigorous enough approach to data collection for this key variable to accept the conclusions presented.

Please submit your revised manuscript by Jul 04 2026 11:59PM. If you will need more time than this to complete your revisions, please reply to this message or contact the journal office at globalpubhealth@plos.org. When you're ready to submit your revision, log on to https://www.editorialmanager.com/pgph/ and select the 'Submissions Needing Revision' folder to locate your manuscript file.

Please include the following items when submitting your revised manuscript:

- A letter that responds to each point raised by the editor and reviewer(s). You should upload this letter as a separate file labeled 'Response to Reviewers'.
- A marked-up copy of your manuscript that highlights changes made to the original version. You should upload this as a separate file labeled 'Revised Manuscript with Track Changes'.
- An unmarked version of your revised paper without tracked changes. You should upload this as a separate file labeled 'Manuscript'.

Guidelines for resubmitting your figure files are available below the reviewer comments at the end of this letter.

As the corresponding author, your ORCID iD is verified in the submission system and will appear in the published article. PLOS supports the use of ORCID, and we encourage all coauthors to register for an ORCID iD and use it as well. Please encourage your coauthors to verify their ORCID iD within the submission system before final acceptance, as unverified ORCID iDs will not appear in the published article. *Only*  the individual author can complete the verification step; PLOS staff *cannot*  verify ORCID iDs on behalf of authors.

We look forward to receiving your revised manuscript.

Kind regards,

Abram L. Wagner, PhD, MPH

Academic Editor

PLOS Global Public Health

Journal Requirements:

If the reviewer comments include a recommendation to cite specific previously published works, please review and evaluate these publications to determine whether they are relevant and should be cited. There is no requirement to cite these works unless the editor has indicated otherwise.

Additional Editor Comments (if provided):

[Note: HTML markup is below. Please do not edit.]

Reviewers' comments:

Reviewer's Responses to Questions

**Comments to the Author**

1. If the authors have adequately addressed your comments raised in a previous round of review and you feel that this manuscript is now acceptable for publication, you may indicate that here to bypass the “Comments to the Author” section, enter your conflict of interest statement in the “Confidential to Editor” section, and submit your "Accept" recommendation.

Reviewer #1: All comments have been addressed

Reviewer #2: (No Response)

Reviewer #3: (No Response)

Reviewer #4: All comments have been addressed

**********

2. Does this manuscript meet PLOS Global Public Health’s publication criteria? Is the manuscript technically sound, and do the data support the conclusions? The manuscript must describe methodologically and ethically rigorous research with conclusions that are appropriately drawn based on the data presented.

Reviewer #1: Yes

Reviewer #2: Partly

Reviewer #3: Partly

Reviewer #4: Yes

**********

3. Has the statistical analysis been performed appropriately and rigorously?

Reviewer #1: I don't know

Reviewer #2: (No Response)

Reviewer #3: I don't know

Reviewer #4: Yes

**********

4. Have the authors made all data underlying the findings in their manuscript fully available (please refer to the Data Availability Statement at the start of the manuscript PDF file)?

The PLOS Data policy requires authors to make all data underlying the findings described in their manuscript fully available without restriction, with rare exception. The data should be provided as part of the manuscript or its supporting information, or deposited to a public repository. For example, in addition to summary statistics, the data points behind means, medians and variance measures should be available. If there are restrictions on publicly sharing data—e.g. participant privacy or use of data from a third party—those must be specified.

Reviewer #1: Yes

Reviewer #2: Yes

Reviewer #3: Yes

Reviewer #4: No

**********

5. Is the manuscript presented in an intelligible fashion and written in standard English?

PLOS Global Public Health does not copyedit accepted manuscripts, so the language in submitted articles must be clear, correct, and unambiguous. Any typographical or grammatical errors should be corrected at revision, so please note any specific errors here.

Reviewer #1: Yes

Reviewer #2: Yes

Reviewer #3: Yes

Reviewer #4: Yes

**********

6. Review Comments to the Author

Please use the space provided to explain your answers to the questions above. You may also include additional comments for the author, including concerns about dual publication, research ethics, or publication ethics. (Please upload your review as an attachment if it exceeds 20,000 characters)

Reviewer #1: This manuscript explores the geospatial disparities in post-pandemic SARS-CoV-2 vaccination in Chile, providing valuable insights that can be used in public health control of infectious diseases.

The proposed framework has potential applicability to research on understanding health behavior in relation to other regions and other diseases, particularly emerging and re-emerging infections.

The authors deserve appreciation for the work that they put into designing the experiments and collecting the data using proper channels and in compliance with ethical considerations.

Certain claims could benefit from references to existing literature, thus lending more credibility to the research process (e.g., Line 56, Line 58, Line 90, Line 103, Line 126).

Please describe the variables used in relation to the categories that they belong to. For example, whether they are discrete or continuous.

More information regarding the statistical test (including any formulas) should be provided under the dedicated section “Statistical analysis”. Spatial autocorrelation analysis needs to be defined properly with sufficient information. All statistical methods used should be described in this section, including the Moran’s Index, Expected index, Z score, and P Value with reference to the context of the study.

The manuscript seems to lack graphical illustrations; while not necessary, it would be better to include a few figures effectively communicating the statistical results.

While not necessary, the authors could discuss how the proposed framework could be adapted to other regions or international contexts as part of future research.

It is better to number the sections on the manuscript to improve its readability and navigation.

Please carefully review the specific points below and revise the manuscript accordingly.

Lines 60-64: Is employment or profession not a considerable factor?

Lines 71-72: Better to restructure this sentence in the past tense.

Line 80: Missing reference.

Line 82: Please revise as “specific population groups who are at more risk”.

Line 83: Change “good coverage” to “sufficient vaccination coverage”?

Line 91: Change “geospatial vaccination distribution post-pandemic SARS-CoV-2 vaccination” to “geospatial distribution of post-pandemic SARS-CoV-2 vaccination”?

Lines 94-95: Please rephrase for clarity.

Line 103: Change to past tense?

Line 114 and more: Links may not be appropriate on the main text. Please check with the journal instructions.

Line 144: Is the phrase “than expected” necessary?

Line 179: Change “by city” to “in terms of the city”?

Table 1: Change “No vaccinated” to “No vaccination” or “Unvaccinated”?

Line 187: Change “masculine” to “men’s” or “male”?

Line 226: Change “in both” to “across both”?

Line 240: Isn't the heterogeneity itself a finding of the study? So, how about phrasing the sentence as follows? "The findings show that there is some heterogeneity in the geospatial distribution of vaccination, while also confirming the hypothesis that this observation is related to socio-demographic factors in both cities."

Lines 245-246: Would it be possible to plot a figure to illustrate this result?

Line 248: Are the correlations strong enough to warrant the use of the word “precisely”? If not, it would be better to use a different word such as "generally".

Line 282: Is the correlation with the number of doses really negative?

Lines 286-287: Change “as described” to “as also described”?

Line 290: Use a more appropriate adjective instead of “deeper”?

Line 315: Change “develop” to “developing”?

Line 319: Change “re-emerge” to “re-emergence”?

Overall, the manuscript is commendable and will be a valuable addition to the relevant literature.

Reviewer #2: General comment

The topic is timely and relevant, and the Chilean context provides a valuable comparative lens given the country’s initially equitable vaccination performance during the pandemic. The study addresses an important gap in understanding post-pandemic vaccination dynamics and offers useful insights into emerging socio-spatial disparities.

Major Comments

1. The "WHO recommendations (reference)" placeholder

In the Introduction (line 80), the sentence reads: "Following the WHO recommendations (reference), since 2023 the Chilean Ministry of Health…" The citation marked "(reference)" remains unfilled. This should be corrected before publication.

2. Disconnect between individual-level data and census-level independent variables

The study collects individual-level data on vaccination (number of doses per person), then assigns socio-demographic independent variables at the census tract level (proportion elderly, educational attainment, overcrowding rate, distance to health center). This is a form of ecological analysis applied to individual-level outcomes, which creates the potential for an ecological fallacy tract-level characteristics may not reflect the individual participant's circumstances. This is not acknowledged in the limitations section, and readers should be cautioned against inferring individual-level causal relationships from these associations. The authors should either address this limitation or, if they consider their analytical approach to sufficiently mitigate it, explain why.

3. Inconsistency in the "Conclusion" column of Tables 2 and 3

There are inconsistencies in the interpretation of spatial autocorrelation results. Several variables with statistically significant p-values (p < 0.05) are described as having “no spatial association,” despite evidence of weak but significant spatial autocorrelation based on Moran’s I. The manuscript would benefit from a clearer distinction between statistical significance (p-value) and strength of association (magnitude of Moran’s I). Even small Moran’s I values, when statistically significant, indicate non-random spatial structure and should be interpreted accordingly (e.g., “weak but significant spatial association”).

4. Interpretation of age-related findings in La Serena/Coquimbo

The interpretation of the age-related findings for La Serena/Coquimbo requires clarification. The results indicate that the proportion of older adults is not spatially associated with vaccination in the total sample or among women, but is associated among men (Table 3; Results section around lines 202–209).

However, the Discussion suggests that clusters of adults younger than 65 receiving more vaccine doses may reflect workplace vaccination access (lines 267–269). This explanation appears speculative, as the study does not include occupational data, workplace vaccination records, or variables capturing access through employment.

This interpretation should either be removed or clearly framed as a hypothesis requiring further investigation. The manuscript should avoid attributing observed spatial patterns to mechanisms not supported by the available data..

5. Sample size and sex-disaggregated analysis

The male sub-samples particularly for Talca (n = 118) are modest. The authors acknowledge the male sample was "scarce in Talca" (Discussion, line 265) but do not address the statistical implications for the sex-disaggregated spatial analyses. With a small and spatially distributed male sample, the absence of significant spatial associations in the Talca male sub-sample may reflect limited statistical power rather than a true absence of spatial patterning. This should be discussed explicitly as a limitation rather than treated symmetrically with the female and total sample results.

6. Two-tier vaccination verification: incomplete manuscript integration

The manuscript describes a two-tier approach for verifying vaccination status (participant-provided documentation and registry confirmation with authorization; lines 129-135). While this strengthens confidence in the data collection process, the reporting remains largely procedural.

The manuscript does not provide a quantitative breakdown of how many participants were verified through each method, nor does it report any instances of missing, unverifiable, or incomplete records.

Given that vaccination status is the primary outcome, greater transparency on data verification and completeness would strengthen the credibility of the findings. A concise quantitative summary of the verification process should be included in the manuscript, rather than only in the response letter.

Minor Comments

• Line 80 (Introduction): The phrase "vaccination coverage is also deteriorating" (present tense) in a description of trends since 2023 is grammatically inconsistent with the surrounding paragraph. Suggest: "vaccination coverage has also been deteriorating."

• Table 1: The total for Talca is listed as n = 336 in the table header, but n = 335 in the Results text (line 161). This discrepancy should be resolved.

• Lines 315-316 (Recommendations): The sentence "Third, develop long-term strategies to improve health literacy and trust-building initiatives may improve the access to vaccination programs" is grammatically incomplete. This may be revised as follows “ Third, long-term strategies to improve health literacy and build trust may enhance access to vaccination programs.”

• Reference 9 is an exact duplicate of Reference 5 (Tesfa GA et al., PLoS One 2023, doi:10.1371/journal.pone.0279399). One should be removed or replaced with the intended citation.

Conclusion and recommendation

Major revision required. The manuscript addresses an important and underexplored topic, and the Chilean context provides a compelling natural experiment for studying the post-pandemic re-emergence of socio-demographic determinants of vaccination. However, the comments outlined above need to be resolved before the paper meets PLOS Global Public Health's publication criteria.

Reviewer #3: Overall: The topic and geospatial approach are relevant and important. However, the authors have not sufficiently justified the approach and some of the objectives are not clear.

lines 61-64: The fragmentation of the referencing here affects the flow of the sentence and can be improved by regrouping the factors with same references so they can be cited together. e.g. age and insurance status [14, 18]. instead of age [14.18], and insurance status [18].

Line 80: No reference included for the WHO recommendation

lines 83-85: This information is important, as it describes post-pandemic vaccine hesitancy, however, I expect also to see why vaccination is still expected or seen to be important in Chile in 2024.

line 91/table 1 and across the paper: It is not clear in this paper what represents the post-pandemic period. This is also important in understanding the vaccine doses reported because some of the doses may have been taken in the pandemic period.

92-93: justification for choosing these cities.

105-108: how were the samples collected, transported and stored? what type of test was done.

130-135: how reliable are these different sources of vaccination records and how do they compare?

The analysis by sex is good. However, the background and objectives did not give any indication of importance or interest in the disparities by sex.

Linea 155-156: what is the reference that informs this cut off for overcrowding?

Table 1: What vaccines constitutes the basic scheme? Please specify the bivalent types or explain briefly in a footnote.

Line 180 (table 1) and other tables: This may not be necessary. Stating sources is more important if the source is from a different study or secondary data.

Tables 2 and 3: what determines if an association is slight spatial association? What are the other categories of spatial associations?

Lines 237-331: In this discussion, I expect a synthesis that compares and contrast with post-pandemic hesitancy in other countries particularly of the some geographic and/or socioeconomic similarities. Where possible, discussion should also highlight similar geospatial approaches from other studies on COVID-19 or other vaccines used in pandemics or epidemics

Reviewer #4: The manuscript addresses a very important issue of public health significance - sustaining equitable vaccine coverage following pandemics. The message is clear, convincing, and backed by sufficient evidence, which is fairly well referenced. Some English language editing of the manuscript is required.

**********

7. PLOS authors have the option to publish the peer review history of their article (what does this mean?). If published, this will include your full peer review and any attached files.

**Do you want your identity to be public for this peer review?**  If you choose “no”, your identity will remain anonymous but your review may still be made public.

For information about this choice, including consent withdrawal, please see our Privacy Policy.

Reviewer #1: **Yes:**  I. M. Hewage

Reviewer #2: No

Reviewer #3: No

Reviewer #4: **Yes:**  Ayodeji O. OLARINMOYE

**********

[NOTE: If reviewer comments were submitted as an attachment file, they will be attached to this email and accessible via the submission site. Please log into your account, locate the manuscript record, and check for the action link "View Attachments". If this link does not appear, there are no attachment files.]

-->Figure Resubmissions:

While revising your submission, we strongly recommend that you use PLOS’s NAAS tool (https://ngplosjournals.pagemajik.ai/artanalysis) to test your figure files. NAAS can convert your figure files to the TIFF file type and meet basic requirements (such as print size, resolution), or provide you with a report on issues that do not meet our requirements and that NAAS cannot fix.

After uploading your figures to PLOS’s NAAS tool - https://ngplosjournals.pagemajik.ai/artanalysis, NAAS will process the files provided and display the results in the "Uploaded Files" section of the page as the processing is complete. If the uploaded figures meet our requirements (or NAAS is able to fix the files to meet our requirements), the figure will be marked as "fixed" above. If NAAS is unable to fix the files, a red "failed" label will appear above. When NAAS has confirmed that the figure files meet our requirements, please download the file via the download option, and include these NAAS processed figure files when submitting your revised manuscript.

# Author comment: Author response to Decision Letter 2

**DOI:** 10.1371/journal.pgph.0006808.r004

1 Jun 2026

Attachment Submitted filename: Point-by-point response.pdf

# Editor report: Decision Letter 2

**DOI:** 10.1371/journal.pgph.0006808.r005

**Authors:** Abram L. Wagner

3 Jun 2026

PGPH-D-25-02096R2

Geospatial disparities in post-pandemic SARS-CoV-2 vaccination: Evidence from Chile beyond the national success

PLOS Global Public Health

Dear Dr. Núñez-Franz,

Thank you for submitting your manuscript to PLOS Global Public Health. After careful consideration, we feel that it has merit but does not fully meet PLOS Global Public Health’s publication criteria as it currently stands. Therefore, we invite you to submit a revised version of the manuscript that addresses the points raised during the review process.

Please submit your revised manuscript by Jul 03 2026 11:59PM. If you will need more time than this to complete your revisions, please reply to this message or contact the journal office at globalpubhealth@plos.org. When you're ready to submit your revision, log on to https://www.editorialmanager.com/pgph/ and select the 'Submissions Needing Revision' folder to locate your manuscript file.

Please include the following items when submitting your revised manuscript:

- A letter that responds to each point raised by the editor and reviewer(s). You should upload this letter as a separate file labeled 'Response to Reviewers'.
- A marked-up copy of your manuscript that highlights changes made to the original version. You should upload this as a separate file labeled 'Revised Manuscript with Track Changes'.
- An unmarked version of your revised paper without tracked changes. You should upload this as a separate file labeled 'Manuscript'.

Guidelines for resubmitting your figure files are available below the reviewer comments at the end of this letter.

As the corresponding author, your ORCID iD is verified in the submission system and will appear in the published article. PLOS supports the use of ORCID, and we encourage all coauthors to register for an ORCID iD and use it as well. Please encourage your coauthors to verify their ORCID iD within the submission system before final acceptance, as unverified ORCID iDs will not appear in the published article. *Only*  the individual author can complete the verification step; PLOS staff *cannot*  verify ORCID iDs on behalf of authors.

We look forward to receiving your revised manuscript.

Kind regards,

Abram L. Wagner, PhD, MPH

Academic Editor

PLOS Global Public Health

**Journal Requirements:**

If the reviewer comments include a recommendation to cite specific previously published works, please review and evaluate these publications to determine whether they are relevant and should be cited. There is no requirement to cite these works unless the editor has indicated otherwise.

Please review your reference list to ensure that it is complete and correct. If you have cited papers that have been retracted, please include the rationale for doing so in the manuscript text, or remove these references and replace them with relevant current references. Any changes to the reference list should be mentioned in the rebuttal letter that accompanies your revised manuscript. If you need to cite a retracted article, indicate the article’s retracted status in the References list and also include a citation and full reference for the retraction notice.

**Additional Editor Comments (if provided):**

I have found you responded well to reviewers. As the editor, I just have one larger question. What do you mean by "structural issues"? The word "structural," in my mind, encompasses distance to clinic - which you list as something which is not structural in page 18 line 320. Essentially, I think you need to slightly discuss structural and social determinants a bit more - or at least define them more precisely - in the discussion. I would think this is a minor change, and I will be able to process your manuscript rapidly with these edits - or a response to me if you do not think edits are warranted.

[Note: HTML markup is below. Please do not edit.]

**Reviewers' comments:**

[NOTE: If reviewer comments were submitted as an attachment file, they will be attached to this email and accessible via the submission site. Please log into your account, locate the manuscript record, and check for the action link "View Attachments". If this link does not appear, there are no attachment files.]

**Figure Resubmissions:**

While revising your submission, we strongly recommend that you use PLOS’s NAAS tool (https://ngplosjournals.pagemajik.ai/artanalysis) to test your figure files. NAAS can convert your figure files to the TIFF file type and meet basic requirements (such as print size, resolution), or provide you with a report on issues that do not meet our requirements and that NAAS cannot fix.

After uploading your figures to PLOS’s NAAS tool - https://ngplosjournals.pagemajik.ai/artanalysis, NAAS will process the files provided and display the results in the "Uploaded Files" section of the page as the processing is complete. If the uploaded figures meet our requirements (or NAAS is able to fix the files to meet our requirements), the figure will be marked as "fixed" above. If NAAS is unable to fix the files, a red "failed" label will appear above. When NAAS has confirmed that the figure files meet our requirements, please download the file via the download option, and include these NAAS processed figure files when submitting your revised manuscript.

# Author comment: Author response to Decision Letter 3

**DOI:** 10.1371/journal.pgph.0006808.r006

5 Jun 2026

Attachment Submitted filename: Point-by-point_response_auresp_3.pdf

# Editor report: Decision Letter 3

**DOI:** 10.1371/journal.pgph.0006808.r007

**Authors:** Abram L. Wagner

19 Jun 2026

Geospatial disparities in post-pandemic SARS-CoV-2 vaccination: Evidence from Chile beyond the national success

PGPH-D-25-02096R3

Dear Núñez-Franz,

We are pleased to inform you that your manuscript 'Geospatial disparities in post-pandemic SARS-CoV-2 vaccination: Evidence from Chile beyond the national success' has been provisionally accepted for publication in PLOS Global Public Health.

Before your manuscript can be formally accepted you will need to complete some formatting changes, which you will receive in a follow up email. A member of our team will be in touch with a set of requests.

Please note that your manuscript will not be scheduled for publication until you have made the required changes, so a swift response is appreciated.

IMPORTANT: The editorial review process is now complete. PLOS will only permit corrections to spelling, formatting or significant scientific errors from this point onwards. Requests for major changes, or any which affect the scientific understanding of your work, will cause delays to the publication date of your manuscript.

If your institution or institutions have a press office, please notify them about your upcoming paper to help maximize its impact. If they'll be preparing press materials, please inform our press team as soon as possible -- no later than 48 hours after receiving the formal acceptance. Your manuscript will remain under strict press embargo until 2 pm Eastern Time on the date of publication. For more information, please contact globalpubhealth@plos.org.

Thank you again for supporting Open Access publishing; we are looking forward to publishing your work in PLOS Global Public Health.

Best regards,

Abram L. Wagner, PhD, MPH

Academic Editor

PLOS Global Public Health

***********************************************************

Reviewer Comments (if any, and for reference):
